# Ligandless Palladium-Catalyzed Direct C-5 Arylation of Azoles Promoted by Benzoic Acid in Anisole

**DOI:** 10.3390/molecules27238454

**Published:** 2022-12-02

**Authors:** Elisabetta Rosadoni, Federico Banchini, Sara Bellini, Marco Lessi, Luca Pasquinelli, Fabio Bellina

**Affiliations:** 1 Dipartimento di Chimica e Chimica Industriale, Università di Pisa, Via Moruzzi 13, 56124 Pisa, Italy; 2 Consorzio C.I.N.M.P.I.S., Via E. Orabona 4, 70125 Bari, Italy

**Keywords:** azoles, direct arylation, C–C coupling, palladium, aromatic solvents, regioselectivity

## Abstract

The palladium-catalyzed direct arylation of azoles with (hetero)aryl halides is nowadays one of the most versatile and efficient procedures for the selective synthesis of heterobiaryls. Although this procedure is, due to its characteristics, also of great interest in the industrial field, the wide use of a reaction medium such as DMF or DMA, two polar aprotic solvents coded as dangerous according to environmental, health, safety (EHS) parameters, strongly limits its actual use. In contrast, the use of aromatic solvents as the reaction medium for direct arylations, although some of them show good EHS values, is poorly reported, probably due to their low solvent power against reagents and their potential involvement in undesired side reactions. In this paper we report an unprecedented selective C-5 arylation procedure involving anisole as an EHS green reaction solvent. In addition, the beneficial role of benzoic acid as an additive was also highlighted, a role that had never been previously described.

## 1. Introduction

(Hetero)arylazoles are key structural cores frequently found in bioactive compounds [1,2,3,4,5,6,7,8,9] and organic functional materials such as liquid crystals [10] and fluorescent dyes [10,11,12,13,14,15]. Due to their widespread applications, the development of straightforward functional group-tolerant synthetic methods that enable selective heteroaromatic elaboration under mild conditions aroused considerable attention.

Among the methods able to functionalize azole scaffolds, the palladium-catalyzed activation of Csp_2_-H bonds represents an attractive strategy for the direct elaboration of their heteroaromatic core [2,4,16,17,18,19,20,21,22,23,24,25,26,27,28], since a pre-activation of both the coupling partners, which is instead required in traditional metal-catalyzed cross-coupling protocols [29] such as Suzuki–Miyaura [30,31,32,33,34,35,36], Migita–Stille [37,38,39,40,41], or Negishi [42,43,44] cross-couplings, is unnecessary. Starting from the pioneering studies by Ohta [45,46] and by Miura [47], synthetic procedures are now available that allow the direct arylation of several azoles, including imidazoles [48,49,50,51,52,53,54,55,56], oxazoles [50,55,57,58,59,60,61,62], thiazoles [10,50,55,63,64], and pyrazoles [50,65,66,67,68,69,70,71]. These reactions are characterized by a wide tolerance towards almost all the main functional groups, and thanks to the ubiquitous presence of C-H bonds they find advantageous application in late-stage functionalization (LSF) protocols useful for introducing molecular diversification in the last step of a synthetic sequence [72,73,74].

However, the presence of different reactive C-H bonds sometimes poses selectivity issues, leading to the formation of regioisomeric monoarylation products and, sometimes, to di- or triarylated azoles as side products.

Although the presence of one or more heteroatoms introduces a “native” differencing of the diverse C-H bonds [75,76], directing the arylation towards the desired position on the heteroaromatic ring is not simple considering the different operational mechanisms that have been suggested over time for these reactions. In fact, mechanistic hypotheses related to the stage of activation of the C-H bond of the catalytic cycle, on which the selectivity of the reaction depends, such as the classic aromatic electrophilic substitution via electrophilic palladation [47,56], the deprotonation–metalation concerted (CMD) [77,78,79,80], or *non*-concerted (*n*-CMD) [81,82], up to the most recent hypothesis of concerted *electrophilic* metalation–deprotonation (*e*-CMD) [83], highlight how there are many factors that influence the real reactivity of the C-H bonds of azoles.

**Figure 1:**
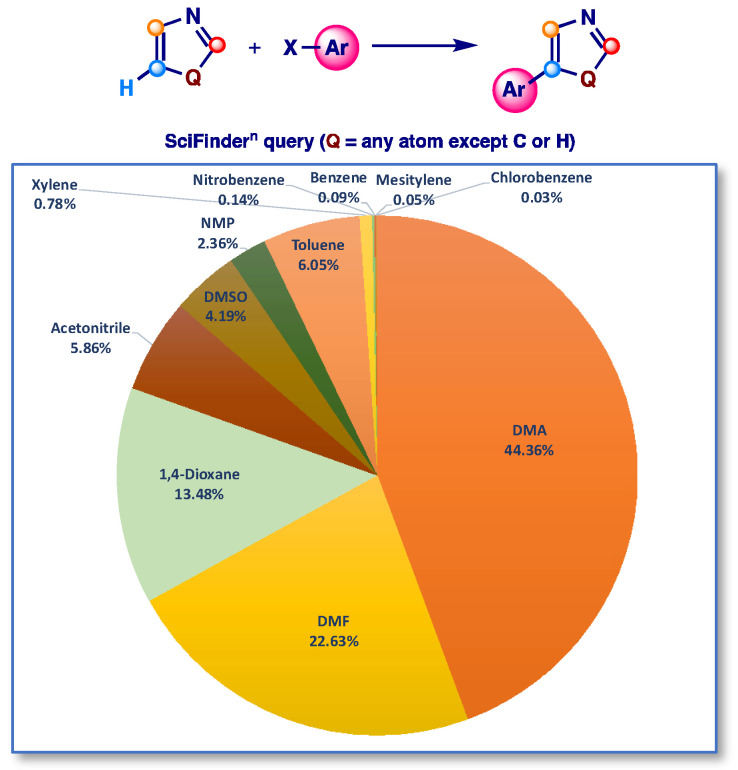
Type of solvents used in Pd-catalyzed direct arylation reactions (from SciFinder^n^, August 2022).

To overcome this relevant issue, research has almost always been oriented towards an optimization of the pre-catalyst/ligand system along with the search for the best inorganic base, while the potential effect of the solvent on the outcome of the coupling has been rarely discussed [84,85,86,87]. In fact, even in cases where a solvent screening has been reported, no comment has been added to justify the different outcome of the arylation. This is much more important if we consider the fact that direct arylation procedures, precisely because of simple operating conditions and high chemoselectivity, can also be very useful in the industrial field. In this regard, an analysis of the solvent used in direct C-5 arylation reactions of 1,3-azoles, carried out by us in August 2022 using SciFinder^n^, clearly shows that the most used solvents are DMA and DMF (over 66% of the total) and that apart from 1,4-dioxane only a little more than 6% of the reactions were conducted in aromatic solvents, therefore different from the polar aprotic ones (Figure 1).

**Figure 2:**
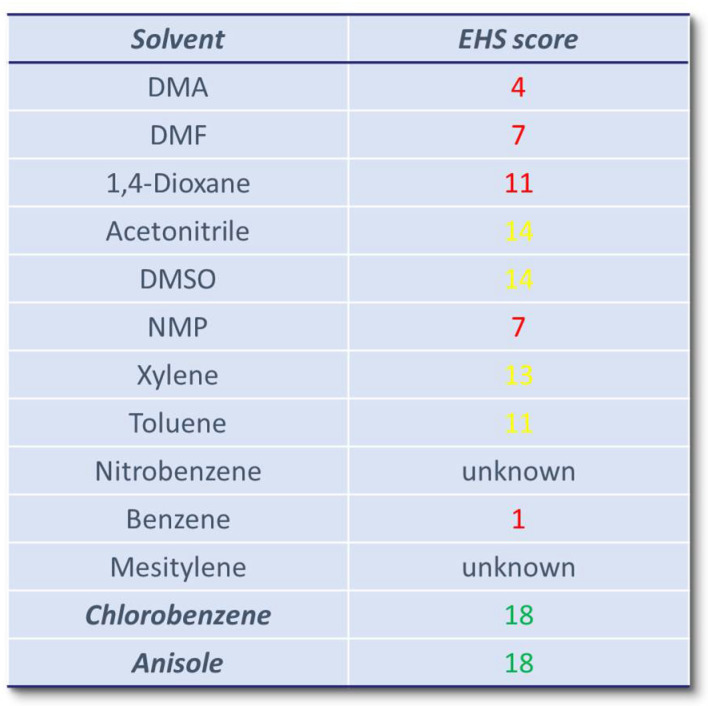
Summarized environmental, health, and safety (EHS) score data label [88,89]. Solvent raws have been color-coded using a traffic light metric: green = recommended (few issues), yellow = problematic (some issues), red = hazardous (major issues). The higher the score, the more favorable the EHS profile of the solvent.

This analysis, despite its limitations, shows that the most widely used solvents, DMA and DMF, are solvents that have been coded as dangerous according to environmental, health, safety (EHS) parameters, while among the “green” EHS solvents only chlorobenzene was employed, while the “green” anisole is totally absent (Figure 2) [88,89]. The sporadic use of aromatic solvents as a reaction medium for direct arylations is probably related to their low solvent power against reagents, and also to their potential involvement in undesired side reactions.

Over recent years we were interested in studies aimed to broaden the substrate scope of the direct functionalization of azoles and, in particular, to develop efficient synthetic protocols for the carbon–carbon bond forming reaction by selective palladium-catalyzed Csp_2_-H bond activation of imidazole derivatives [48,50,52,53,54,55,56,90,91].

**Scheme 1:**
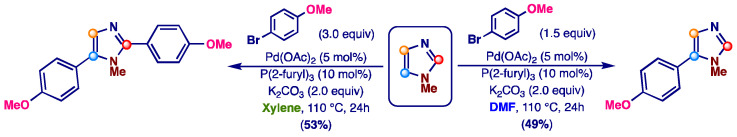
Influence of reaction solvent on the Pd-catalyzed direct arylation of 1-methylimidazole with 4-bromoanisole [48,90].

During these studies, we discovered that the outcome of the Pd-catalyzed arylation of imidazoles with aryl bromides is deeply influenced by the nature of the reaction solvent. Specifically, while it is well known that the Pd-catalyzed direct arylation of imidazoles with aromatic halides selectively leads to C-5 monoarylation products when polar aprotic solvents such as DMF (or DMA) are used as a reaction medium [23,47,50,52,56,90], we have recently observed the preferential formation of C-2,5 double arylation products simply by using xylene as the reaction solvent (Scheme 1) [48].

**Scheme 2:**
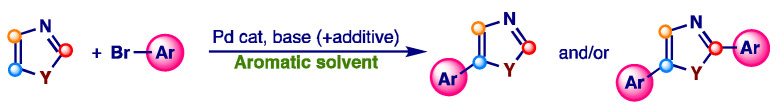
Pd-catalyzed direct arylation of 1,3-azoles with aryl bromides using aromatic solvents.

Intrigued by the influence of aromatic solvents on the reactivity of Csp_2_-H heteroaromatic bonds and by the good EHS parameters of aromatic solvents, as mentioned above, we started a study devoted to evaluating the influence of the aromatic solvents on the efficiency and the selectivity of the direct arylation of imidazoles and other azoles. In particular, in this paper we will discuss the possibility of achieving C-5 selectivity in aromatic solvents, studying how the ratio between mono- and diarylated products changes in function of the nature of the aromatic solvent and of the electronic characteristic of aromatic bromide (Scheme 2).

## 2. Results and Discussion

As discussed in the Introduction, the results obtained using xylene as an aromatic solvent for the direct arylation of imidazoles showed a substantially lower selectivity towards monoarylation, combined with a greater efficiency of coupling when electron-rich aromatic bromides, such as 4-bromoanisole or 4-bromoaniline, were used as coupling partners [48].

**Scheme 3:**
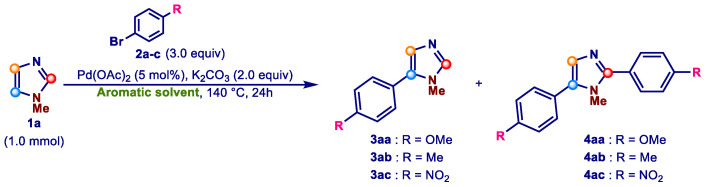
First screening of aromatic solvents for the Pd-catalyzed direct arylation of 1-methylimidazole **1a** with aryl bromides **2a–c**.

To verify the possible influence of the aromatic solvent on the efficiency and selectivity of azole arylation, we started the study by evaluating the outcome of the coupling of 1-methyl-1*H*-imidazole (**1a**), chosen as the model azole, with aromatic bromides in four different aromatic solvents: xylenes, anisole, chlorobenzene, and nitrobenzene. These solvents have been selected because they all have a boiling point equal to or greater than 140 °C, the temperature at which we have decided to conduct the initial screening. As coupling partners we chose three aromatic bromides, selected for their different electronic characteristics: 4-bromoanisole (**2a**) (Hammett’s σ_p_ = −0.27 [92]), 4-bromotoluene (**2b**) (Hammett’s σ_p_ = −0.17 [92]), and 1-bromo-4-nitrobenzene (**2c**) (Hammett’s σ_p_ = 0.78 [92]). The screening was carried out using 5 mol% of Pd(OAc)_2_ as the palladium pre-catalyst, 2.0 equiv of K_2_CO_3_ as the base, 1.0 mmol of **1a**, 3.0 equiv of bromides **2a–c**, in 5.0 mL of the aromatic solvent at 140 °C for 24 h (Scheme 3). To better highlight the possible effect of individual solvents, we decided to carry out the screening under ligandless conditions.

The results of the screening are summarized in Table 1, where the GLC yields of **3** and **4** along with the selectivity of the monoarylation vs. the diarylation are reported.

Examination of the data given in Table 1 shows that the use of anisole as the reaction solvent allowed us to obtain selectively the monoarylated 5-arylimidazole **3**, regardless of the electronic nature of the aromatic bromides (Entries 10–12, Table 1). On the contrary, the selectivity of **3** vs. **4** when the other three aromatic solvents were used seems to be clearly influenced by the substituent present on the aromatic ring of bromides **2**. In fact, in nitrobenzene (Entries 1–3, Table 1), chlorobenzene (Entries 4–6, Table 1), and xylenes (Entries 7–9, Table 1), the highest selectivity was observed with 1-bromo-4-nitrobenzene (**2c**) (Entries 3, 6, and 9, Table 1), and the lowest when 4-bromoanisole (**2a**) (Entries 1, 4, and 7, Table 1) was used as a coupling partner. It is also worth mentioning that the selectivity of **3** vs. **4** is reversed using anisole as the reaction solvent (Entries 10–12, Table 1).

Intrigued by the fact that by using anisole as the reaction solvent we observed selective arylation toward the monoarylated **3aa–ac** products regardless of the electronic nature of the bromides **2a–c** (despite the **1**:**2** molar ratio being 1:3), and that the trend in selectivity as a function of the electronic nature of the aryl bromide **2** was inverse to that found with the other three aromatic solvents, we decided to start a new screening using anisole as the reaction solvent, and choosing 1-bromo-4-nitrobenzene (**2c**) because it had given the worst selectivity in the same solvent.

To our surprise, the first results of the screening in anisole showed that the selectivity of the reaction, conducted under the exact experimental conditions shown in Table 1, was dependent on the commercial origin of anisole (Table 2).

**Figure 3:**
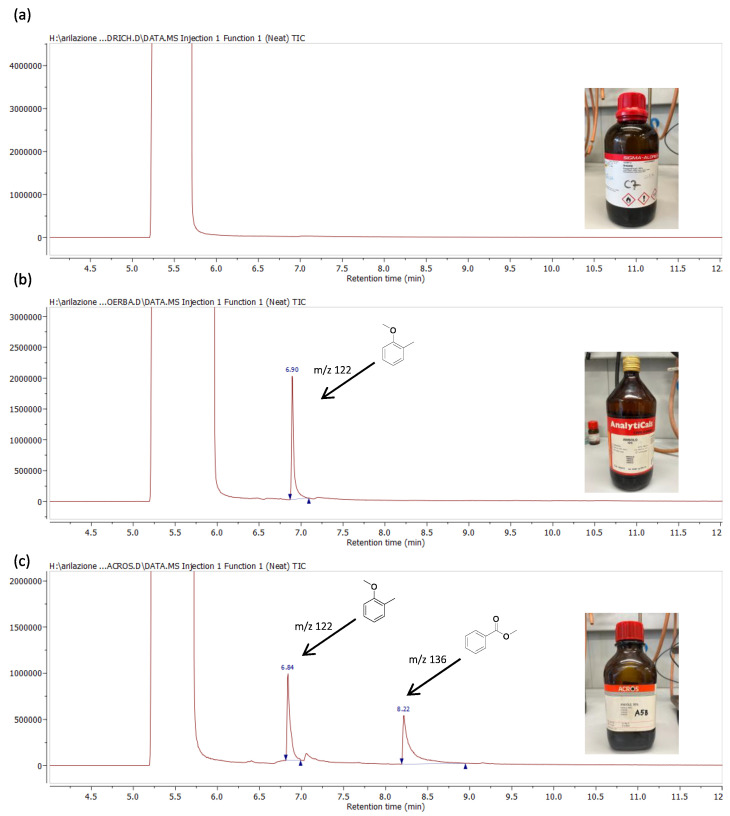
GLC-EIMS of anisole from Acros (**a**), Carlo Erba (**b**), and Sigma-Aldrich (**c**).

To understand these selectivity results a GLC analysis of the different reaction solvents was carried out. In detail, GLC-MS analysis of small aliquots of commercial anisole from the three suppliers (Table 2) showed that in AC anisole methyl benzoate was present as an impurity, in addition to 2-methylanisole (Figure 3).

The presence of this ester only in the AC anisole, together with the greater selectivity observed in this specific solvent, has led us to think that this compound or, much more likely, the benzoate analogue that can be formed in a basic environment, not strictly anhydrous (the solvents were simply deaerated with argon), may have an important role in the efficiency and selectivity of the reaction. It is in fact well known that aliphatic carboxylic acids [93], such as pivalic acid or its salts, can effectively promote the selective C-5 arylation of imidazoles and other azoles by means of a CMD mechanism [78,79,80,94]. In this circumstance, the alkyl carboxylate acts as a base-shuttle between the inorganic base and the catalytically active palladium complexes, entering the coordination sphere of the transition metal and favoring the extraction of the proton in the activation step of the heteroaromatic C-H bond.

**Scheme 4:**
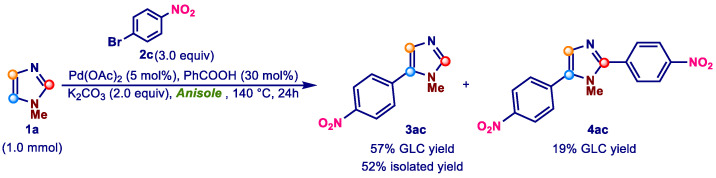
Direct arylation of 1-methylimidazole (**1a**) with 1-bromo-4-nitrobenzene (**2c**) in SA anisole using benzoic acid as an additive.

Therefore, assuming that the same role could be effectively played by benzoic acid in an aromatic solvent such as anisole, we conducted a first test using SA anisole as solvent, the purest of the three batches examined (Scheme 4).

As shown in Scheme 4, the addition of 30 mol% benzoic acid was beneficial to reaction outcome. In fact, **3ac** was recovered in 52% isolated yield, and the selectivity was substantially identical to that found when AC anisole was used (compare Scheme 4 with Entry 12, Table 1).

With the aim of further increasing the selectivity and yield of the monoarylation reaction, we performed an additional screening using only 1.5 equiv of 1-bromo-4-nitrobenzene (**2c**) (Table 3).

As can be seen from Entry 1, Table 3, by reducing the aryl bromide to 1.5 equiv the conversion of **1a** remained high and the percentage of the diarylation product (**4ac**) decreased, so **3ac** was obtained with a GLC yield of 75% (72% isolated). A reduction in catalytic loading to 2.5 mol% (Entry 2, Table 3) resulted in a slight decrease in conversion of **1a** and GLC yield of **3ac**. Lowering the reaction temperature from 140 to 120 °C (Entry 3, Table 3) led to a decrease in conversion of **1a** and yield of **3ac**. Carrying out the reaction in xylenes as the reaction solvent (Entry 4, Table 3) gave a high conversion of precursor **1a** but also a higher amount of the diarylation product (**4ac**) than the reaction conducted in anisole, giving **3ac** in 63% yield. When other additives were tried, i.e., pivalic acid and phenol (Entries 5 and 6, Table 3), worse results in terms of **3ac** yield were obtained. Tests were also carried out with different kinds of phosphines as palladium ligands (tris(o-tolyl)phosphine, tri(2-furyl)phosphine, tricyclohexylphosphine, and dppf), but in all cases the **3ac** yields were lower than that observed under ligandless conditions (Table S1, Supporting Information).

The satisfactory result obtained in the preparation of **3ac** from **1a** and **2c** under the experimental conditions summarized in Entry 1, Table 3 prompted us to extend this methodology to the selective synthesis of several 5-arylazoles in anisole as the reaction solvent.

**Scheme 5:**
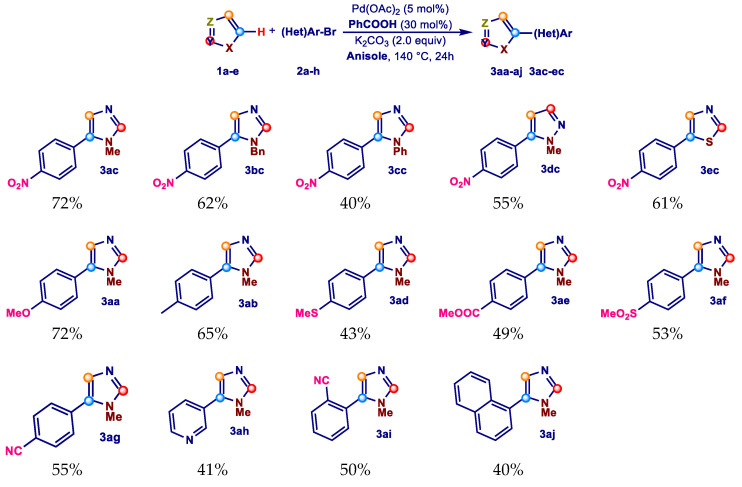
Selective Pd-catalyzed direct arylation of azoles **1a–e** with aryl bromides **2a–h**.

In detail, performing the reaction in the presence of 5 mol% Pd(OAc)_2_, 30 mol% benzoic acid, and 2.0 equiv K_2_CO_3_ in 5 mL of anisole under ligandless conditions, we were able to recover the required 5-aryl substituted derivatives **3ac–3ec** and **3aa–3aj** in 40–72% isolated yield after 24 h at 140 °C (Scheme 5).

As can be seen from Scheme 5, the coupling also works efficiently when 1-benzyllimidazole (**1b**) and 1-phenylimidazole (**1c**) were used as partner of coupling with 1-bromo-4-nitrobenzene (**2c**), giving the desired products **3bc** and **3cc** in 62 and 40% isolated yield. Satisfactory results were also obtained with 1-methylpyrazole (**2d**) and thiazole (**2e**), giving the products **3dc** and **3ec** with isolated yields of 55 and 61%, respectively.

Subsequent tests were performed by varying the nature of the aryl bromide. In particular, the electron-rich bromides 4-bromoanisole (**2a**), 4-bromotoluene (**2b**), and 4-bromothioanisole (**2d**) gave the respective 5-aryl products **3aa**, **3ab**, and **3ad** in 72, 65, and 43% isolated yields. Good results were also obtained with the electron-poor methyl 4-bromobenzoate (**2e**), 1-bromo-4-(methylsulfonyl)benzene (**2f**), 4-bromobenzonitrile (**2g**), and 3-bromopyridine (**2h**), which resulted in the respective products **3ae–3ah** with isolated yields of 49, 53, 55, and 41%. In the end, the procedure was tested with the sterically hindered bromides 2-bromobenzonitrile (**2i**) and 1-bromonaphthalene (**2j**), and it proved to be effective; products **3ai** and **3aj**, in fact, were obtained with yields of 50 and 40%.

**Figure 4:**
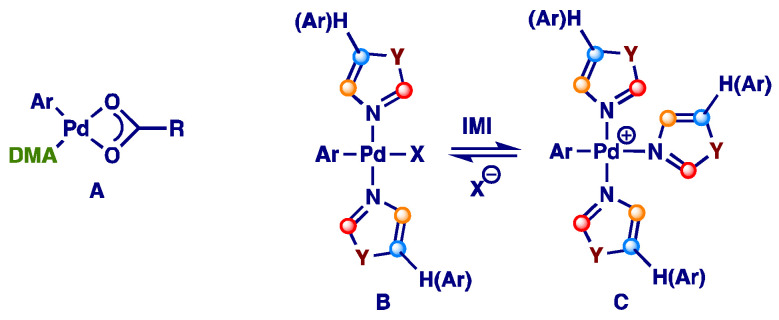
Supposed Pd(II)-catalyzed direct arylation mechanism in aromatic solvents. **A**: DMA solvate Pd complex; **B** and **C**: structures of azole-ligated arylpallaium intermediates.

Further studies are required to elucidate the operative mechanism in aromatic solvents. However, while it was demonstrated that in DMA a solvate complex with palladium **A** seems to play an important role in the catalytic cycle [95], in an aromatic solvent having poorer coordinating ability azole-ligated organo-palladium intermediates **B** and **C** could be the active catalytic species (Figure 4) [96].

Moreover, the fact that the observed reactivity of azoles parallels that of classical electrophilic aromatic substitution (EAS) [97], and that higher efficiency was obtained when benzoic acid was added, an *electrophilic* concerted metalation–deprotonation (*e*-CMD) mechanistic pathway [83] seems to be the most plausible among the various mechanistic hypotheses formulated for the palladium-catalyzed direct arylation of azoles.

## 3. Materials and Methods

### 3.1. General Information

Melting points were recorded on a hot-stage microscope (Reichert, Wien, Austria, Thermovar). Precoated silica gel PET foils (Sigma-Aldrich, St. Louis, MI, USA) were used for TLC analyses. GLC-FID analyses were performed on a Dani (Milan, Italy) GC 1000 chromatograph equipped with a PTV injector, using an Agilent (Santa Clara, USA) J&W DB-1 column (15 m × 0.25 mm × 0.25 μm) and recorded with a Dani DDS 1000 data station. GLC-MS analyses were recorded with an Agilent 6890N gas chromatograph interfaced with an Agilent MS5973 mass detector, using an Agilent J&W DB-5ms (30 m × 0.25 mm × 0.25 μm) column. Purifications by flash chromatography were performed using Merck 60 silica gel. ^1^H-NMR and ^13^C-NMR spectra were recorded at 400 and 100 MHz, respectively, with a Jeol (Tokyo, Japan) 400 spectrometer referring chemical shifts to the residual solvent signal. The following notation was used to report NMR spectra: s = singlet, d = doublet, dd = double doublet, t = triplet, dt = double triplet, q = quadruplet. All the commercially available reagents and solvents were used as received.

### 3.2. Procedure for the Screening of the aromatic solvents for the Pd-Catalyzed 5-Arylation of 1-Methyl-1H-imidazole (**1a**) with Aryl Bromides **2a–c**

Pd(OAc)_2_ (11.2 mg, 0.05 mmol), aryl bromide (**2a–c**) (3.0 mmol), if solid, and K_2_CO_3_ (276 mg, 2.0 mmol) were placed in a reaction vessel. The reaction vessel was fitted with a silicon septum, evacuated, and backfilled with argon. This sequence was repeated twice. The selected deaerated solvent (5 mL), aryl bromide **2** (3.0 mmol), if a liquid, and 1-methyl-1*H*-imidazole (**1a**) (82 mg, 80 µL, 1.0 mmol) were then added successively under a stream of argon by syringe. The resulting mixture was stirred under argon for 24 h at 140 °C. After cooling to room temperature, the crude reaction mixture was diluted with DCM and AcOEt, biphenyl was added as internal standard, and the resulting mixture was analyzed by GLC and GC–MS. The results of this screening are summarized in Table 1 and Table 2.

### 3.3. Procedure for the Screening of the Reaction Conditions for the Pd-Catalyzed 5-Arylation of 1-Methyl-1H-imidazole (**1a**) with 1-Bromo-4-Nitrobenzene (**2c**) in Anisole (SA)

Pd(OAc)_2_ (11.2 mg, 0.05 mmol), additive (30 mol%), 1-bromo-4-nitrobenzene (**2c**) (303 mg, 1.5 mmol), and K_2_CO_3_ (276 mg, 2.0 mmol) were placed in a reaction vessel. The reaction vessel was fitted with a silicon septum, evacuated, and backfilled with argon. This sequence was repeated twice. The selected deaerated solvent (5 mL) and 1-methyl-1*H*-imidazole (**1a**) (82 mg, 80 µL, 1.0 mmol), were then added successively under a stream of argon by syringe. The resulting mixture was stirred under argon for 24 h at the selected temperature. After cooling to room temperature, the crude reaction mixture was diluted with DCM and AcOEt, biphenyl was added as internal standard, and the resulting mixture was analyzed by GLC and GC–MS. Table 3 summarizes the results of this screening.

### 3.4. Procedure for the Screening of the Reaction Scope for the Pd-Catalyzed 5-Arylation of Azoles (**1a–e**) with Aryl Bromides (**2a–j**) in Anisole (SA)

Pd(OAc)_2_ (11.2 mg, 0.05 mmol), benzoic acid (36 mg, 0.30 mmol), azole **1** (1 mmol), if solid, aryl bromide **2** (1.5 mmol), if solid, and K_2_CO_3_ (276 mg, 2.0 mmol) were placed in a reaction vessel. The reaction vessel was fitted with a silicon septum, evacuated, and backfilled with argon. This sequence was repeated twice. Anisole (5 mL), azole **1** (1 mmol), if liquid, and aryl bromide **2**, if liquid, were then added successively under a stream of argon by syringe. The resulting mixture was stirred under argon for 24 h at 140 °C. After cooling to room temperature, the crude reaction mixture was diluted with DCM and AcOEt. The resulting mixture was analyzed by GLC and GC–MS and concentrated under reduced pressure and the residue purified by flash chromatography on silica gel. This procedure was used to prepare compounds **3ac–3aj** and **3bc–3ec**. For the product **3aa** and **3ab** the reaction was carried out with 1.0 mmol of the aryl bromide **2** and 1.5 mmol of 1-Methyl-1*H*-imidazole (**1a**). The results are summarized in Scheme 5.

#### 3.4.1. 1-methyl-5-(4-nitrophenyl)-1H-imidazole (**3ac**)

The crude reaction product obtained by the coupling reaction of 1-methyl-1*H*-imidazole (**1a**) with 1-bromo-4-nitrobenzene (**2c**) (Scheme 5) was purified by flash chromatography on silica gel with a mixture of DCM and MeOH (93:7) as eluent to give **3ac** as a yellow-orange solid, (145 mg, 72%), m.p. 165–167 °C (lit. m.p. 169–171 °C) [50]. ESI-MS *m*/*z* 204 [M+H]^+^. EI-MS, *m*/*z* (%): 203 (100), 173 (19), 130 (17), 103 (16), 89 (32). ^1^H NMR (400 MHz, CDCl_3_) δ 8.32–8.28 (m, 2H), 7.61–7.55 (m, 3H), 7.27 (s, 1H), 3.76 (s, 3H). The spectral properties of this compound are in agreement with those previously reported [50].

#### 3.4.2. 1-benzyl-5-(4-nitrophenyl)-1H-imidazole (**3bc**)

The crude reaction product obtained by the coupling reaction of 1-benzyl-1*H*-imidazole (**1b**) with 1-bromo-4-nitrobenzene (**2c**) (Scheme 5) was purified by flash chromatography on silica gel with a mixture of DCM and MeOH (95:5) as eluent to give **3bc** as an orange solid, (173 mg, 62%), m.p. 108–109 °C (lit. m.p. 106–108 °C) [98]. ESI-MS *m*/*z* 280 [M+H]^+^. EI-MS, *m*/*z* (%): 91 (100), 279 (46), 65 (10), 280 (9), 92 (8). ^1^H NMR (400 MHz, CDCl_3_) δ 8.24–8.16 (m, 2H), 7.67 (s, 1H), 7.49–7.41 (m, 2H), 7.40–7.24 (m, 4H), 7.04–6.97 (m, 2H), 5.23 (s, 2H). The spectral properties of this compound are in agreement with those previously reported [98].

#### 3.4.3. 5-(4-nitrophenyl)-1-phenyl-1H-imidazole (**3cc**)

The crude reaction product obtained by the coupling reaction of 1-phenyl-1*H*-imidazole (**1c**) with 1-bromo-4-nitrobenzene (**2c**) (Scheme 5) was purified by flash chromatography on silica gel with a mixture of toluene and ethyl acetate (80:20) as eluent to give **3cc** as a yellow solid, (106 mg, 40%), m.p. 155–156 °C (lit. m.p. 162–164 °C) [99]. ESI-MS *m*/*z* 266 [M+H]^+^. EI-MS, *m*/*z* (%): 265 (100), 191 (19), 266 (17), 165 (16), 192 (15). ^1^H NMR (400 MHz, CDCl_3_) δ 8.15–8.07 (m, 2H), 7.77 (d, *J* = 1.0 Hz, 1H), 7.48–7.43 (m, 4H), 7.30–7.26 (m, 2H), 7.25–7.17 (m, 2H). The spectral properties of this compound are in agreement with those previously reported [100].

#### 3.4.4. 1-methyl-5-(4-nitrophenyl)-1H-pyrazole (**3dc**)

The crude reaction product obtained by the coupling reaction of 1-methyl-1*H*-pyrazole (**1d**) with 1-bromo-4-nitrobenzene (**2c**) (Scheme 5) was purified by flash chromatography on silica gel with a mixture of dichloromethane and acetone (98:2) as eluent to give **3dc** as a yellow solid, (112 mg, 55%), m.p. 85–86 °C (lit. m.p. 75–77 °C) [50]. ESI-MS *m*/*z* 204 [M+H]^+^. EI-MS, *m*/*z* (%): 203 (100), 173 (20), 204 (12), 103 (11), 89 (10). ^1^H NMR (400 MHz, CDCl3) δ 8.37–8.29 (m, 2H), 7.64–7.59 (m, 2H), 7.57 (d, J = 2.0 Hz, 1H), 6.43 (d, J = 2.0 Hz, 1H), 3.95 (s, 3H). The spectral properties of this compound are in agreement with those previously reported [50].

#### 3.4.5. 5-(4-nitrophenyl)thiazole (**3ec**)

The crude reaction product obtained by the coupling reaction of thiazole (**1e**) with 1-bromo-4-nitrobenzene (**2c**) (Scheme 5) was purified by flash chromatography on silica gel with a mixture of toluene and ethyl acetate (80:20) as eluent to give **3ec** as a yellow solid, (126 mg, 61%), m.p. 142–143 °C (lit. m.p. 139–141 °C) [50]. ESI-MS *m*/*z* 207 [M+H]^+^. EI-MS, *m*/*z* (%): 206 (100), 176 (26), 148 (20), 133 (27), 89 (53). ^1^H NMR (400 MHz, CDCl_3_) δ 8.88 (s, 1H), 8.33–8.22 (m, 2H), 8.23 (s, 1H), 7.77–7.72 (m, 2H). The spectral properties of this compound are in agreement with those previously reported [50].

#### 3.4.6. 5-(4-methoxyphenyl)-1-methyl-1H-imidazole (**3aa**)

The crude reaction product obtained by the coupling reaction of 1-methyl-1*H*-imidazole (**1a**) with 1-bromo-4-methoxybenzene (**2a**) (Scheme 5) was purified by flash chromatography on silica gel with a mixture of DCM and MeOH (96:4) as eluent to give **3aa** as a white solid, (135 mg, 72%), m.p. 77–78 °C (lit. m.p. 73–75 °C) [50]. ESI-MS *m*/*z* 189 [M+H]^+^. EI-MS, *m*/*z* (%): 188 (100), 173 (82), 145 (17), 189 (12), 174 (10). ^1^H NMR (400 MHz, CDCl_3_) δ 7.49 (s, 1H), 7.34–7.27 (m, 2H), 7.03 (s, 1H), 7.00–6.93 (m, 2H), 3.85 (s, 2H), 3.63 (s, 2H). The spectral properties of this compound are in agreement with those previously reported [50].

#### 3.4.7. 1-methyl-5-(p-tolyl)-1H-imidazole (**3ab**)

The crude reaction product obtained by the coupling reaction of 1-methyl-1*H*-imidazole (**1a**) with 1-bromo-4-methylbenzene (**2b**) (Scheme 5) was purified by flash chromatography on silica gel with a mixture of DCM and MeOH (95:5) as eluent to give **3ab** as a yellow oil, (112 mg, 65%). ESI-MS *m*/*z* 173 [M+H]^+^. EI-MS, *m*/*z* (%): 172 (100), 171 (17), 130 (16), 144 (14), 173 (13). ^1^H NMR (400 MHz, CDCl_3_) δ 7.51 (s, 1H), 7.30–7.22 (m, 5H), 7.07 (d, J = 1.1 Hz, 1H), 3.65 (s, 3H), 2.40 (s, 3H). The spectral properties of this compound are in agreement with those previously reported [50].

#### 3.4.8. 1-methyl-5-(4-(methylthio)phenyl)-1H-imidazole (**3ad**)

The crude reaction product obtained by the coupling reaction of 1-methyl-1*H*-imidazole (**1a**) with 1-(4-bromophenyl)(methyl)sulfane (**2d**) (Scheme 5) was purified by flash chromatography on silica gel with a mixture of DCM and MeOH (94:6) as eluent to give **3ad** as a white solid, (88 mg, 43%), m.p. 71–73 °C. ESI-MS *m*/*z* 205 [M+H]^+^. EI-MS, *m*/*z* (%): 204 (100), 189 (63), 205 (13), 190 (8), 162 (7). ^1^H NMR (400 MHz, CDCl_3_) δ 7.48 (s, 1H). 7.30–7.25 (m, 4H), 7.05 (s, 1H), 3.62 (s, 3H), 2.49 (s, 3H). ^13^C NMR (100 MHz, CDCl_3_) δ 139.12, 138.60, 132.99, 128.81, 128.04, 126.53, 126.41, 32.55, 15.63. Elemental analysis calcd. for C_11_H_12_N_2_S: C, 64.67; H, 5.92; N, 13.71; found C, 64.71; H, 5.91; N, 13.69.

#### 3.4.9. methyl 4-(1-methyl-1H-imidazol-5-yl)benzoate (**3ae**)

The crude reaction product obtained by the coupling reaction of 1-methyl-1*H*-imidazole (**1a**) with methyl 4-bromobenzoate (**2e**) (Scheme 5) was purified by flash chromatography on silica gel with a mixture of DCM and MeOH (96:4) as eluent to give **3ae** as a white solid, (106 mg, 49%), m.p. 128–130 °C. ESI-MS *m*/*z* 217 [M+H]^+^. EI-MS, *m*/*z* (%): 216 (100), 185 (78), 217 (14), 89 (13), 130 (12). ^1^H NMR (400 MHz, CDCl_3_) δ 8.12–8.07 (m, 2H), 7.55 (s, 1H), 7.50–7.46 (m, 2H), 7.20 (s, 1H), 3.94 (s, 3H), 3.72 (s, 3H). The spectral properties of this compound are in agreement with those previously reported [101].

#### 3.4.10. 1-methyl-5-(4-(methylsulfonyl)phenyl)-1H-imidazole (**3af**)

The crude reaction product obtained by the coupling reaction of 1-methyl-1*H*-imidazole (**1a**) with 1-bromo-4-(methylsulfonyl)benzene (**2f**) (Scheme 5) was purified by flash chromatography on silica gel with a mixture of DCM and MeOH (95:5) as eluent to give **3af** as a white solid, (130 mg, 53%), m.p. 189–190 °C. ESI-MS *m*/*z* 237 [M+H]^+^. EI-MS, *m*/*z* (%): 236 (100), 173 (33), 157 (26), 89 (18), 130 (17). ^1^H NMR (400 MHz, CDCl_3_) δ 8.01–7.97 (m, 2H), 7.61–7.58 (m, 2H), 7.57 (s, 1H), 7.21 (s, 1H), 3.73 (s, 3H), 3.09 (s, 3H). ^13^C NMR (100 MHz, CDCl_3_) δ 140.65, 139.50, 135.47, 131.68, 130.03, 128.67, 128.07, 44.62, 33.02. Elemental analysis calcd. for C_11_H_12_N_2_O_2_S: C, 55.92; H, 5.12; N, 11.86; found C, 55.97; H, 5.13; N, 11.88.

#### 3.4.11. 4-(1-methyl-1H-imidazol-5-yl)benzonitrile (**3ag**)

The crude reaction product obtained by the coupling reaction of 1-methyl-1*H*-imidazole (**1a**) with 4-bromobenzonitrile (**2g**) (Scheme 5) was purified by flash chromatography on silica gel with a mixture of DCM and MeOH (95:5) as eluent to give **3ag** as a yellow solid, (101 mg, 55%), m.p. 146–147 °C (lit. m.p. 148–151 °C) [50]. ESI-MS *m*/*z* 184 [M+H]^+^. EI-MS, *m*/*z* (%): 183 (100), 155 (14), 184 (13), 128 (11), 114 (10). ^1^H NMR (400 MHz, CDCl_3_) δ 7.74–7.70 (m, 2H), 7.57 (s, 1H), 7.53–7.50 (m, 2H), 7.22 (s, 1H), 3.73 (s, 3H). The spectral properties of this compound are in agreement with those previously reported [50].

#### 3.4.12. 3-(1-methyl-1H-imidazol-5-yl)pyridine (**3ah**)

The crude reaction product obtained by the coupling reaction of 1-methyl-1*H*-imidazole (**1a**) with 3-bromopyridine (**2h**) (Scheme 5) was purified by flash chromatography on silica gel with a mixture of DCM and MeOH (95:5) as eluent to give **3ah** as a pale green oil, (65 mg, 41%), ESI-MS *m*/*z* 160 [M+H]^+^. EI-MS, *m*/*z* (%): 160 (11), 159 (100), 158 (9), 131 (32), 104 (10). ^1^H NMR (400 MHz, CDCl_3_) δ 8.61–8.58 (m, 1H), 8.55–8.51 (m, 1H), 7.67–7.63 (m, 1H), 7.50 (s, 1H), 7.34–7.29 (m, 1H), 7.09 (s, 1H), 3.62 (s, 3H). The spectral properties of this compound are in agreement with those previously reported [90].

#### 3.4.13. 2-(1-methyl-1H-imidazol-5-yl)benzonitrile (**3ai**)

The crude reaction product obtained by the coupling reaction of 1-methyl-1*H*-imidazole (**1a**) with 2-bromobenzonitrile (**2i**) (Scheme 5) was purified by flash chromatography on silica gel with a mixture of DCM and MeOH (95:5) as eluent to give **3ai** as a yellow solid, (91 mg, 50%), m.p. 153–155 °C (lit. m.p. 156–158 °C) [102]. ESI-MS *m*/*z* 184 [M+H]^+^. EI-MS, *m*/*z* (%): 183 (100), 155 (31), 129 (27), 156 (20), 182 (19). ^1^H NMR (400 MHz, CDCl_3_) δ 7.79 (ddd, J = 7.7, 1.4, 0.6 Hz, 1H), 7.67 (td, J = 7.7, 1.4 Hz, 1H), 7.60 (s, 1H), 7.51 (td, J = 7.7, 1.2 Hz, 1H), 7.44 (ddd, J = 7.7, 1.2, 0.6 Hz, 1H), 7.24 (s, 1H), 3.64 (s, 3H). The spectral properties of this compound are in agreement with those previously reported [102].

#### 3.4.14. 1-methyl-5-(naphthalen-1-yl)-1H-imidazole (**3aj**)

The crude reaction product obtained by the coupling reaction of 1-methyl-1*H*-imidazole (**1a**) with 1-bromonaphthalene (**2j**) (Scheme 5) was purified by flash chromatography on silica gel with a mixture of DCM and MeOH (95:5) as eluent to give **3aj** as an orange solid, (94 mg, 45%), m.p. 140–144 °C. ESI-MS *m*/*z* 209 [M+H]^+^. EI-MS, *m*/*z* (%): 208 (100), 207 (36), 166 (20), 153 (19), 180 (15). ^1^H NMR (400 MHz, CDCl_3_) δ 7.95–7.89 (m, 2H), 7.67–7.63 (m, 2H), 7.56–7.43 (m, 4H), 7.16 (s, 1H), 3.42 (s, 3H). The spectral properties of this compound are in agreement with those previously reported [103].

## 4. Conclusions

In conclusion, in this work, we developed a simple and efficient ligandless Pd-catalyzed selective C-5 direct arylation of imidazoles and other azoles with aryl bromides, using anisole as the reaction solvent. In fact, with the aim of verifying the possible influence of aromatic solvents on the efficiency and selectivity arylation of azoles, having good environmental, health, safety (EHS) parameters, we started a study with 1-methyl-1*H*-imidazole (**1a**), chosen as the model azole, and aromatic bromides in four different solvents: xylenes, anisole, chlorobenzene, and nitrobenzene. After this preliminary screening, we discovered a high C-5 selectivity in anisole; specifically, when the anisole used was from a specific supplier, and we observed that in this solvent ethyl benzoate was present as an impurity. This has led us to think that this compound or, much more likely, the benzoate analogue that can be formed in a basic environment, not strictly anhydrous, may have an important role on the efficiency and selectivity of the reaction. Therefore, assuming that the same role could be effectively played by benzoic acid, we conducted a test with the addition of 30 mol% benzoic acid and this was beneficial to reaction outcome, increasing the monoarylation selectivity. So, after a final fine-tuning of the conditions, we found that by performing the reaction in the presence of Pd(OAc)_2_ as a pre-catalyst, benzoic acid as an additive, and K_2_CO_3_ as a base in anisole, we recovered several 5-aryl substituted azoles in 72–40% isolated yield after 24 h at 140 °C. Further studies on the interesting role of aromatic solvents in direct arylation reactions and on their role in the reaction mechanism are undergoing.

## Figures and Tables

**Table 1:** Screening of aromatic solvents ^1^.

Entry	Solvent	2	R	3 Yield (%) ^2^	4 Yield (%) ^2^	Selectivity ^3^
1	Nitrobenzene	**2a**	OMe	30	33	0.9
2	Nitrobenzene	**2b**	Me	67	20	3.4
3	Nitrobenzene	**2c**	NO_2_	65	18	3.6
4	Chlorobenzene	**2a**	OMe	41	35	1.2
5	Chlorobenzene	**2b**	Me	27	46	0.6
6	Chlorobenzene	**2c**	NO_2_	60	8	7.5
7	Xylenes	**2a**	OMe	20	38	0.5
8	Xylenes	**2b**	Me	53	36	1.5
9	Xylenes	**2c**	NO_2_	64	8	8.0
10	Anisole	**2a**	OMe	40	6	6.7
11	Anisole	**2b**	Me	60	12	5.0
12	Anisole	**2c**	NO_2_	56 (43)	24	2.3

^1^ The reactions were carried out using imidazole **1a** (1.0 mmol), aryl bromides **2a–c** (3.0 equiv), Pd(OAc)_2_ (5 mol%), K_2_CO_3_ (2.0 equiv) in 5.0 mL of deaerated aromatic solvent at 140 °C for 24 h, for all reactions the conversion of **1a** is greater than 95%. ^2^ GLC yield using biphenyl as internal standard; isolated yield is given in brackets. ^3^ The selectivity is expressed as the ratio of GLC yield of **3** to GLC yield of **4**.

**Table 2:** Direct arylation of 1-methylimidazole **1a** with 1-bromo-4-nitrobenzene (**2c**) in anisole from different suppliers ^1^.

Entry	Supplier	Cat. N.	3ac Yield (%) ^2^	4ac Yield (%) ^2^	Selectivity ^3^
1	Acros (AC)	15,392	56	24	2.3
2	Carlo Erba (CE)	422,677	49	40	1.2
3	Sigma-Aldrich (SA)	123,266	47	30	1.6

^1^ The reactions were carried out using imidazole **1a** (1.0 mmol), aryl bromides **2a–c** (3.0 equiv), Pd(OAc)_2_ (5 mol%), K_2_CO_3_ (2.0 equiv) in 5.0 mL of deaerated anisole at 140 °C for 24 h, for all reactions the conversion of **1a** is greater than 95%. ^2^ GLC yield using biphenyl as internal standard. ^3^ The selectivity is expressed as the ratio of GLC yield of **3** to GLC yield of **4**.

**Table 3:** Synthesis of 5-arylimidazole **3c** by direct arylation of 1-methylimidazole (**1a**) with 1-bromo-4-nitrobenzene (**2c**) in SA anisole using benzoic acid as additive.^1^

Entry	Acidic Additive	1a Conv (%) ^2^	3ac Yield (%) ^3^	4ac Yield (%) ^3^
1	Benzoic acid	93	75(72)	14
2 ^4^	Benzoic acid	87	71	10
3 ^5^	Benzoic acid	56	35	1
4 ^6^	Benzoic acid	90	63	22
5	Pivalic acid	75	35	12
6	Phenol	79	47	11

^1^ Unless otherwise stated, the reactions were carried out using imidazole **1a** (1.0 mmol), 1-bromo-4-nitrobenzene (**2c**) (1.5 equiv), Pd(OAc)_2_ (5 mol%), PhCOOH (30 mol%), K_2_CO_3_ (2.0 equiv) in 5.0 mL of deaerated SA anisole at 140 °C for 24 h. ^2^ GLC conversion of **1a** vs. biphenyl ^3^ GLC yield using biphenyl as internal standard; isolated yield is given in brackets. ^4^ The reaction was carried out using 2.5 mol% Pd(OAc)_2_. ^5^ The reaction was carried out at 120 °C. ^6^ A commercial mixture of *o-* and *p*-xylene (xylenes) was used as the reaction solvent.

## Data Availability

Not applicable.

## Supplementary Materials

The following supporting information can be downloaded at: https://www.mdpi.com/article/10.3390/molecules27238454/s1, Table S1: Screening of the ligands, Figures S1–S16: NMR spectra of compounds **3ac–3ec** and **3aa–3aj**.
