# Supplementary material for: Ligandless Palladium-Catalyzed Direct C-5 Arylation of Azoles Promoted by Benzoic Acid in Anisole
Source: Molecules. 2022 Dec 2;27(23):8454. doi: 10.3390/molecules27238454 (PMC9735507; doi:10.3390/molecules27238454)
Supplement: Supplementary file 1 [file molecules-27-08454-s001.zip › molecules-2062196-supplementary.pdf]

Table S1. Screening of the ligands

Entry	ligand	**1a** Conv (%)[2]	**3ac** Yield (%)[3]	**4ac** Yield (%)[3]
1	$P(o\text{-}Tol)_3$	75	54	22
2	$P(2\text{-}furyl)_3$	93	63	27
3	$P(cycloexyl)_3$	95	57	35
4	dppf	81	51	14

[1] The reactions were carried out using 1-methylimidazole (**1a**) (1.0 mmol), 1-bromo-4-nitrobenzene (**2c**) (1.5 equiv), $Pd(OAc)_2$ (5 mol%), PhCOOH (30 mol%), K_2CO_3 (2.0 equiv) in 5.0 mL of deaerated SA anisole at 140 °C for 24 h. [2] GLC conversion of **1** vs. biphenyl [3] GLC yield using biphenyl as internal standard.

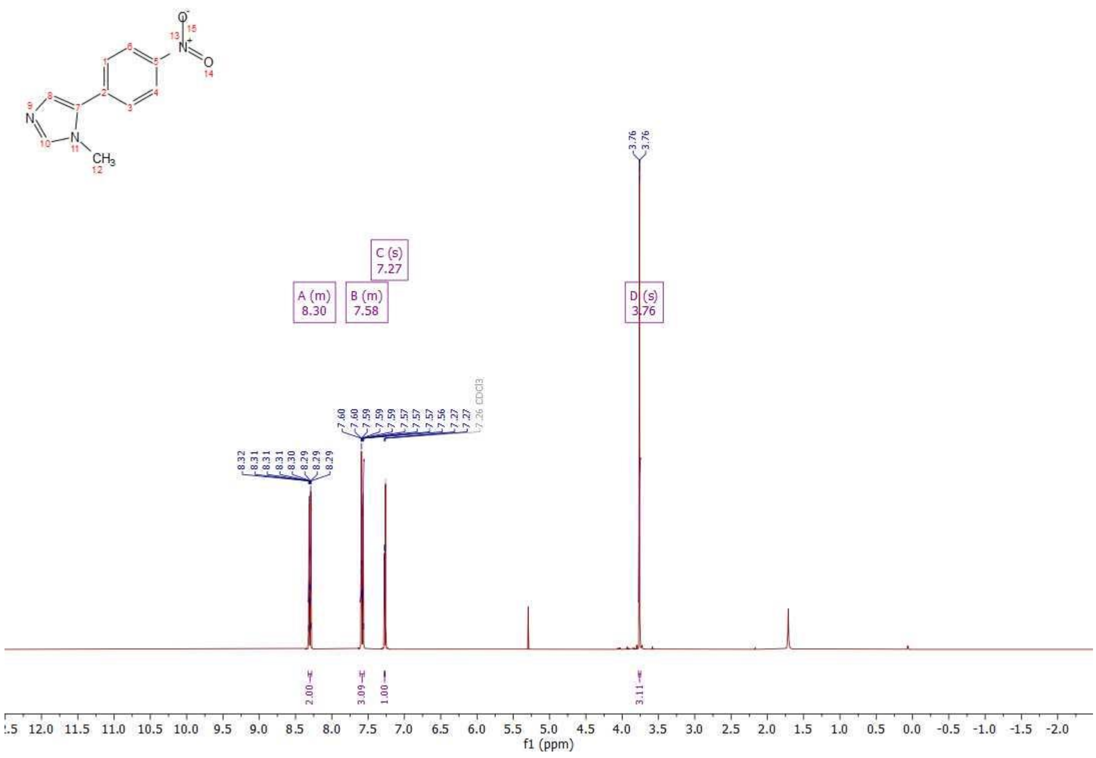

Figure S1 ^{1}H NMR 5-(4-nitrophenyl)-1-phenyl-1H-imidazole (**3ac**)

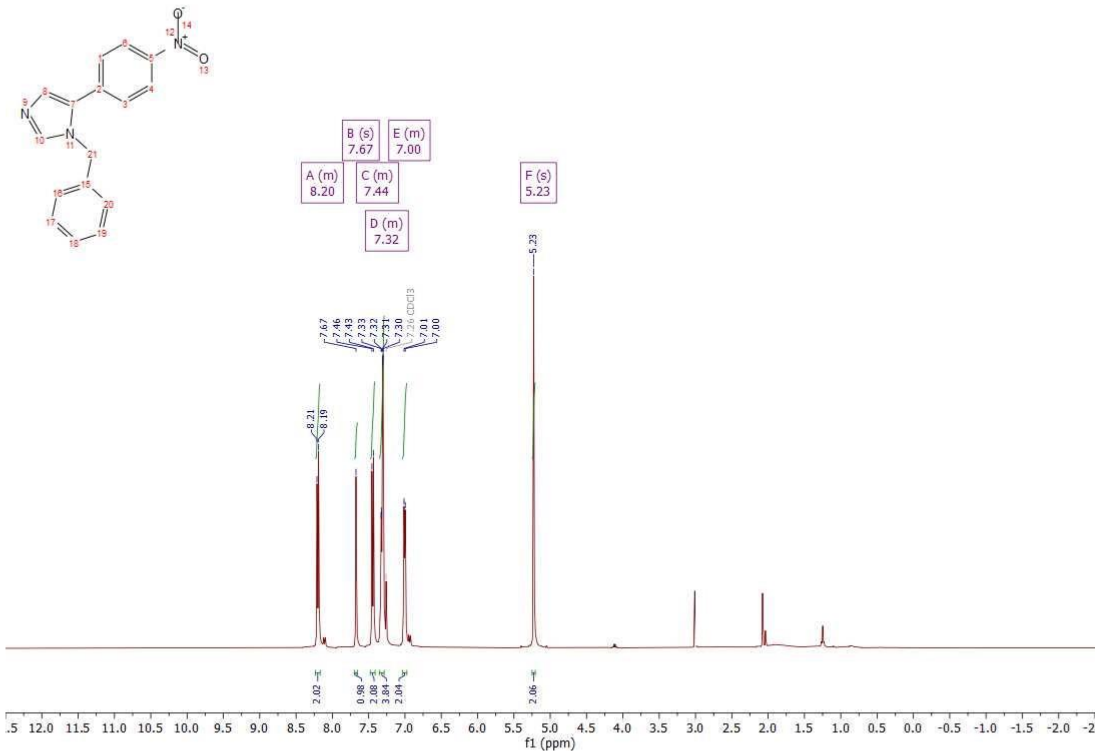

Figure S2 ^{1}H NMR 1-benzyl-5-(4-nitrophenyl)-1H-imidazole (**3bc**)

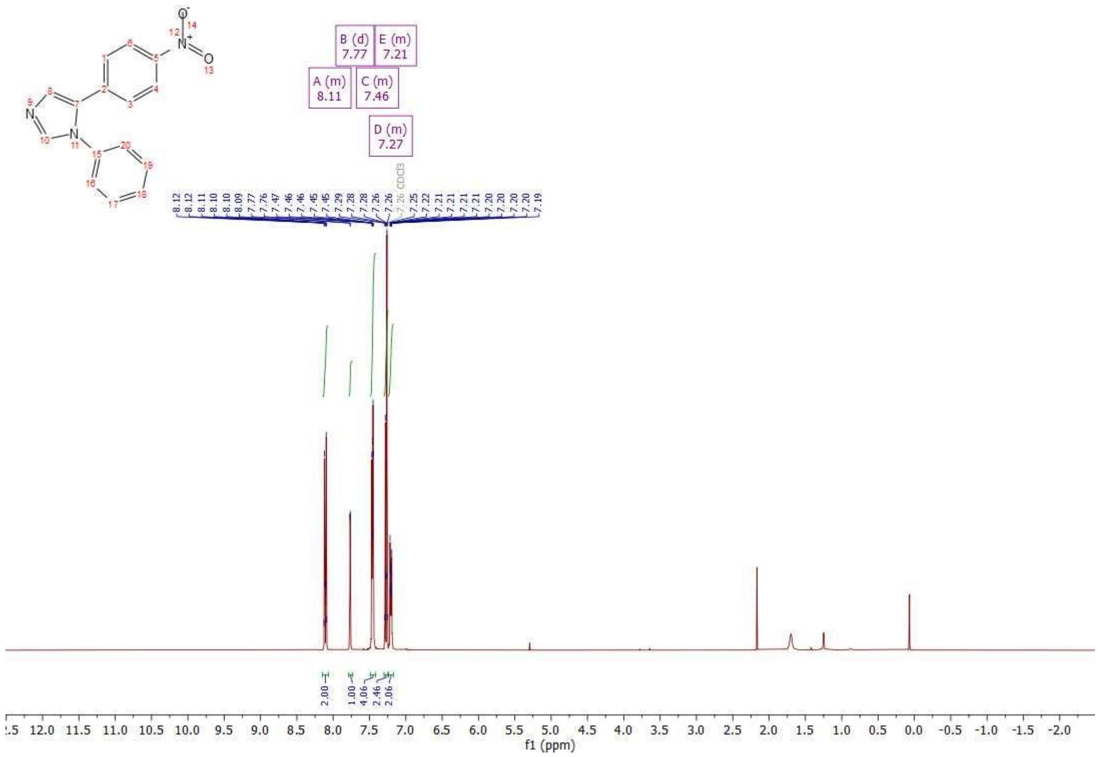

Figure S3 ^{1}H NMR 5-(4-nitrophenyl)-1-phenyl-1H-imidazole (**3cc**)

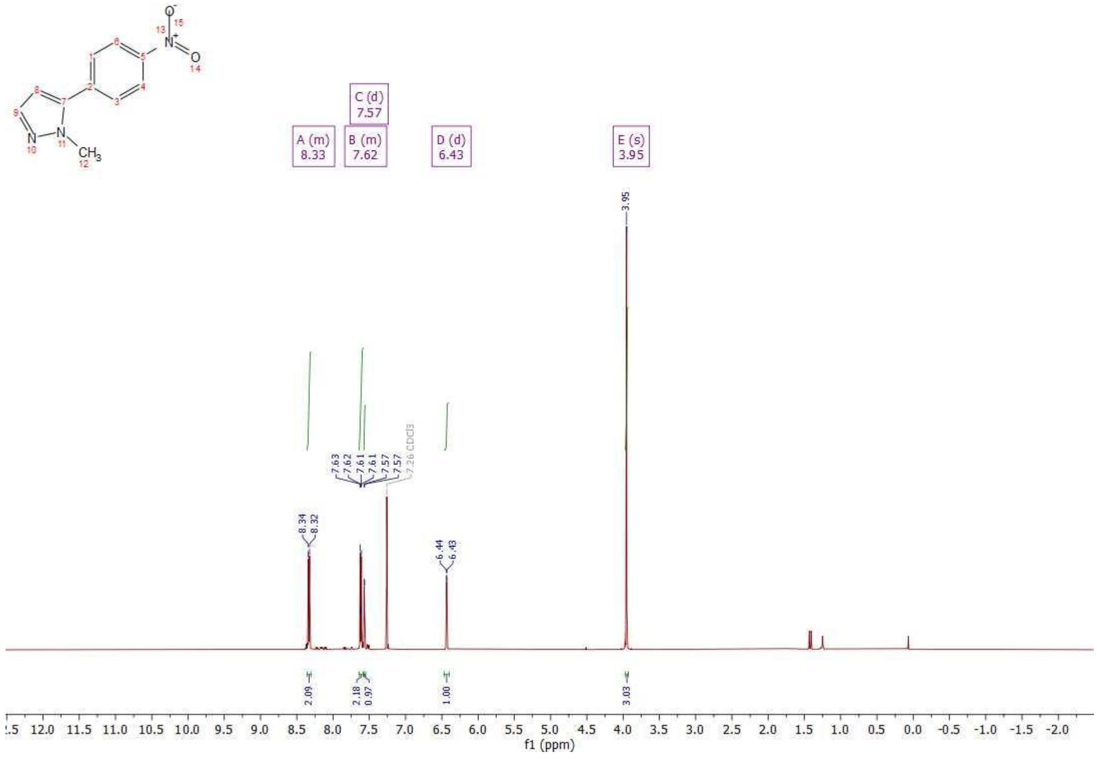

Figure S4 ^{1}H NMR 1-methyl-5-(4-nitrophenyl)-1H-pyrazole (**3dc**)

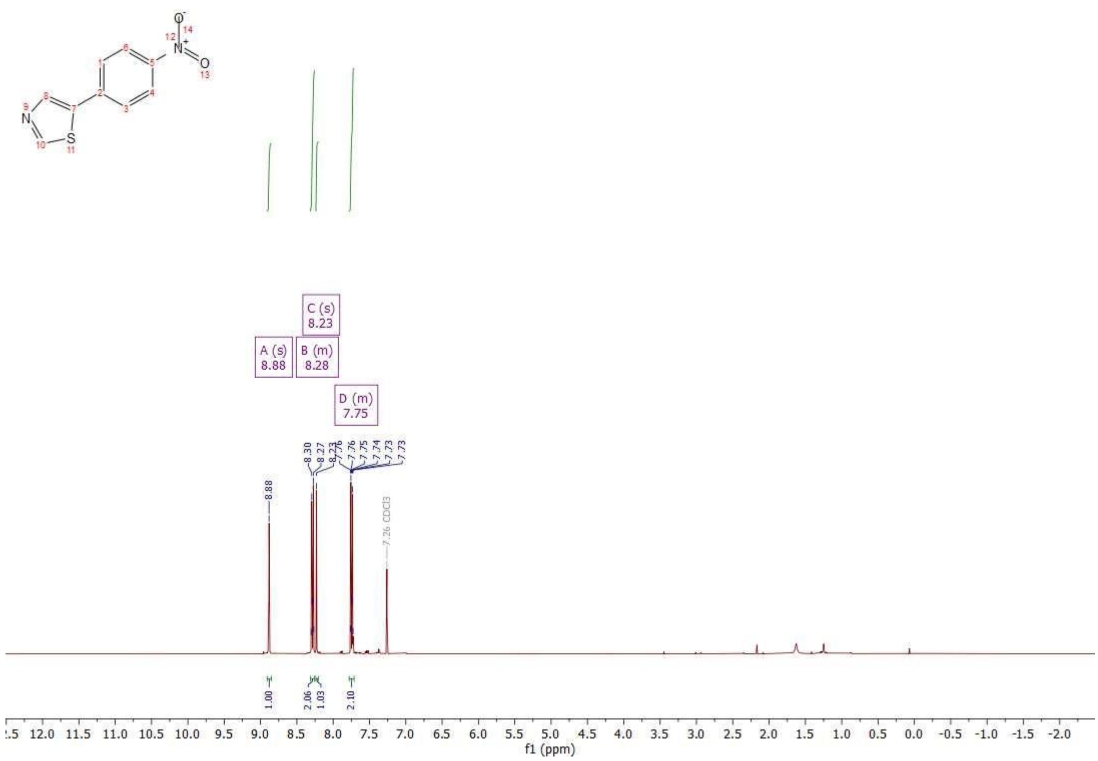

Figure S5 ^{1}H NMR 5-(4-nitrophenyl)thiazole (**3ec**)

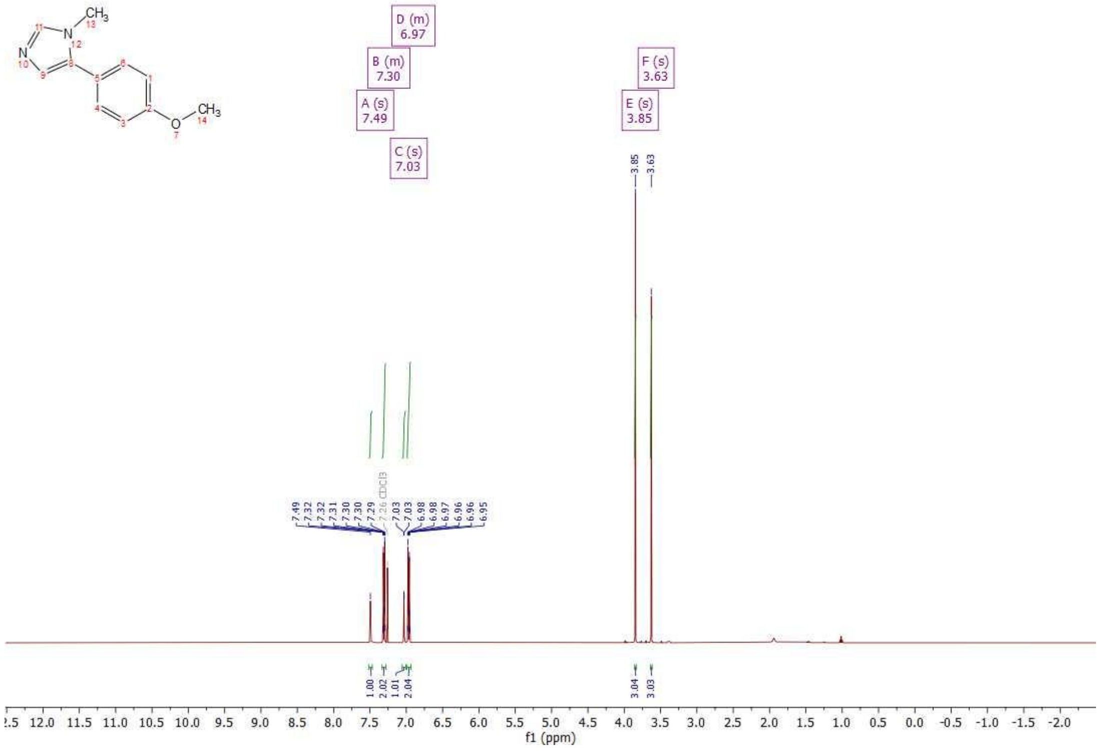

Figure S6 ^{1}H NMR 5-(4-methoxyphenyl)-1-methyl-1H-imidazole (**3aa**)

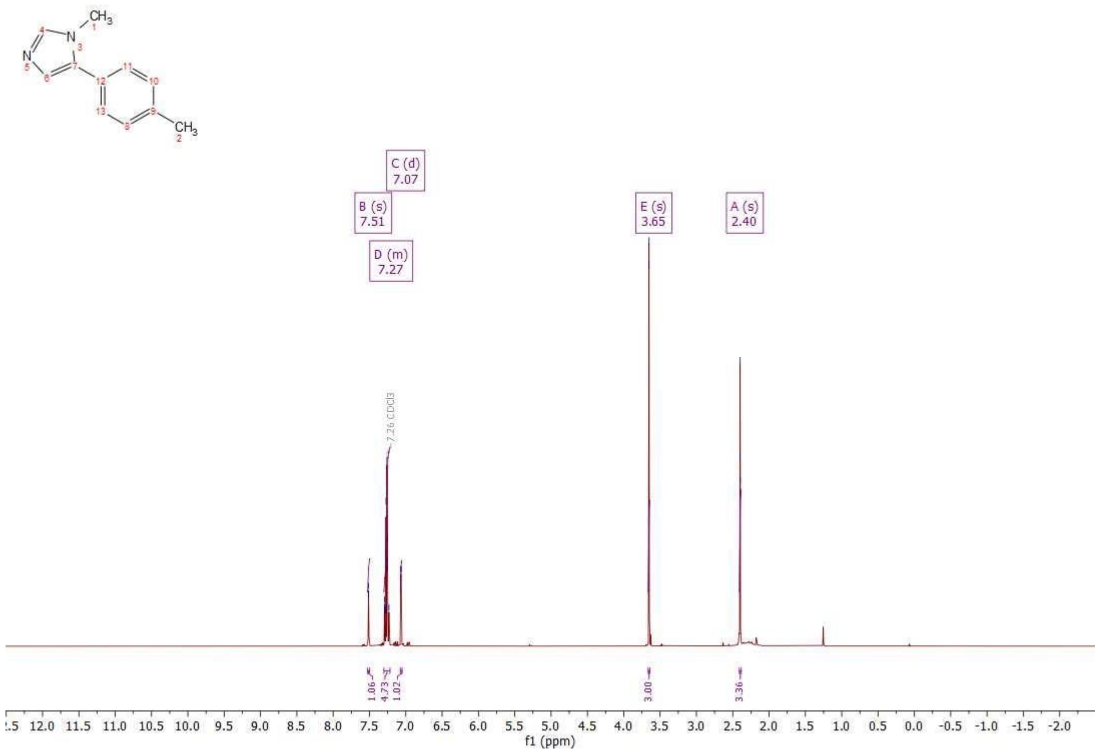

Figure S7 ^{1}H NMR 1-methyl-5-(p-tolyl)-1H-imidazole (**3ab**)

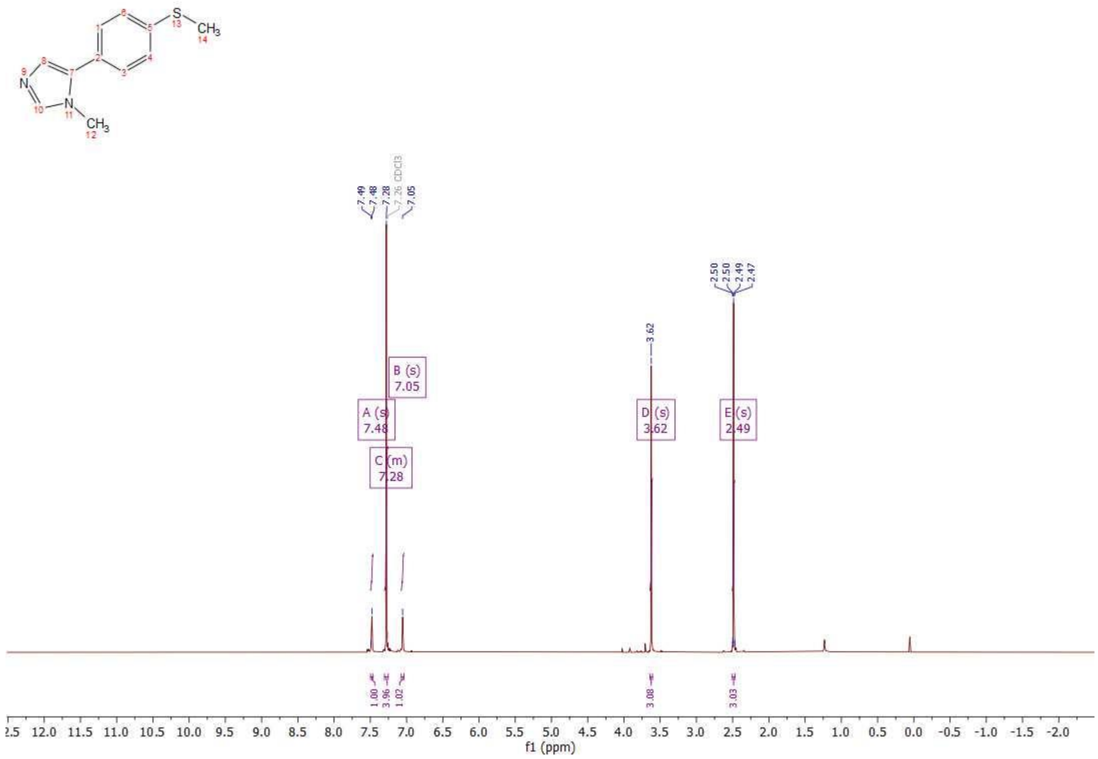

Figure S8 ^{1}H NMR 1-methyl-5-(4-(methylthio)phenyl)-1H-imidazole (**3ad**)

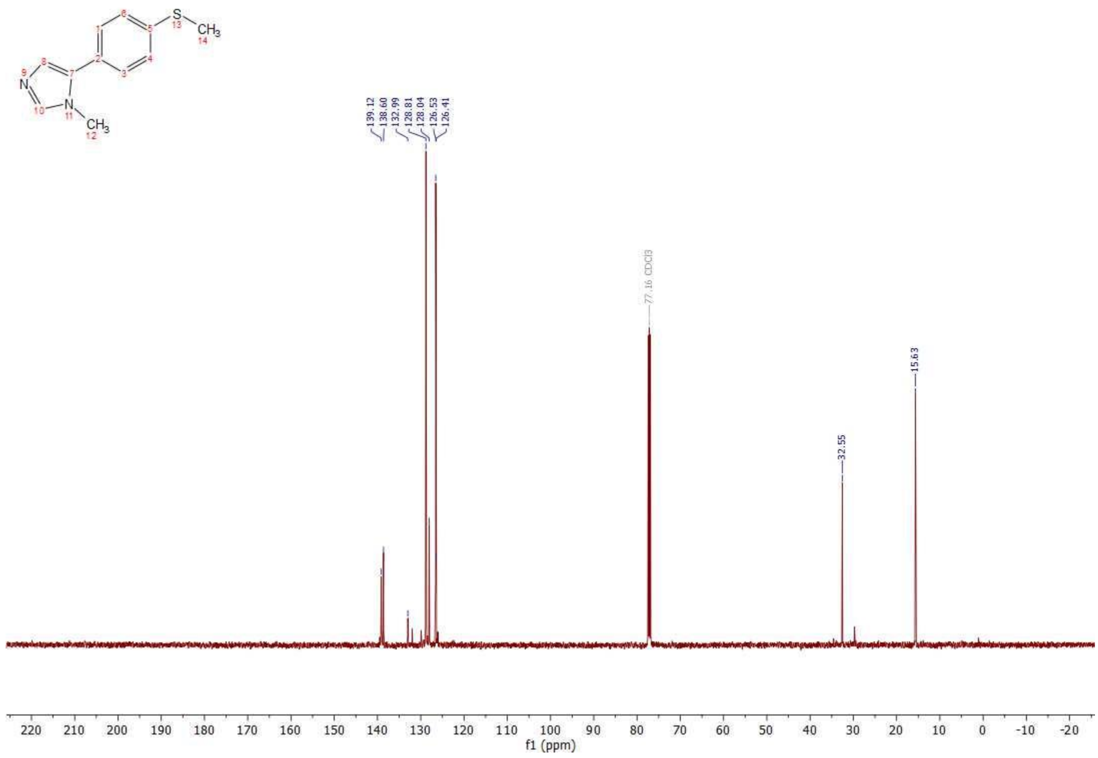

Figure S9 ^{13}C NMR 1-methyl-5-(4-(methylthio)phenyl)-1H-imidazole (**3ad**)

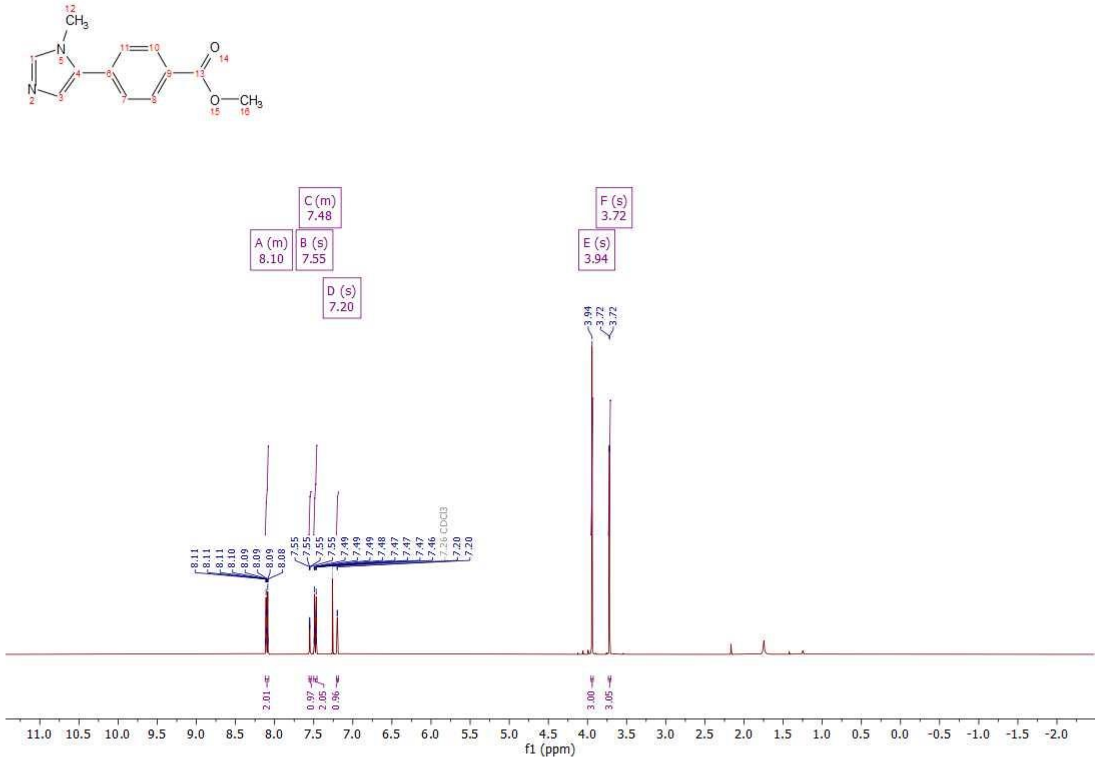

Figure S10 ^{1}H NMR 1-methyl-5-(4-(methylsulfonyl)phenyl)-1H-imidazole (**3ae**)

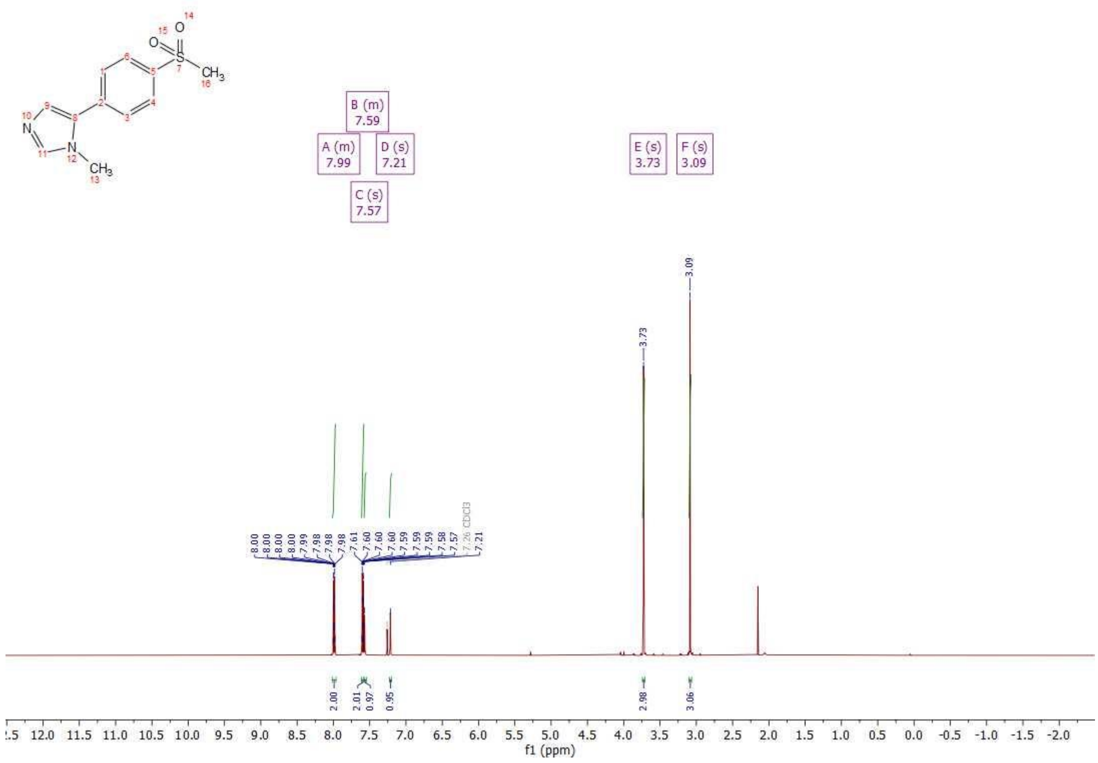

Figure S11 ^{1}H NMR 1-methyl-5-(4-(methylsulfonyl)phenyl)-1H-imidazole (**3af**)

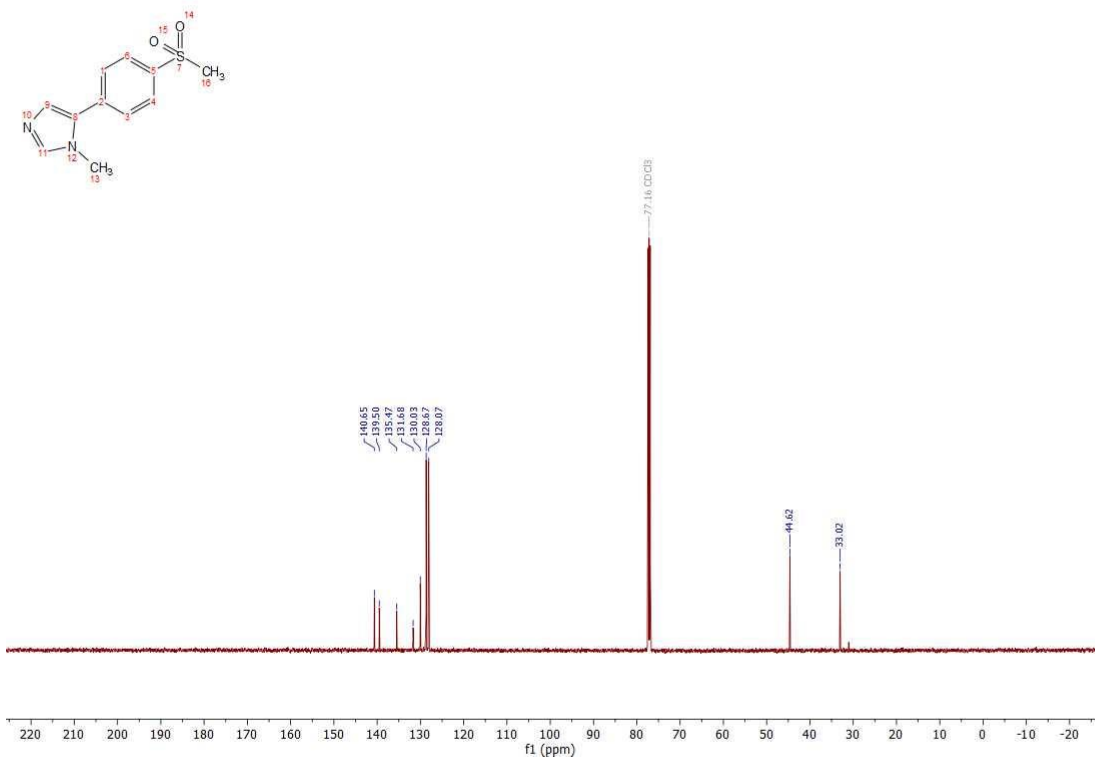

Figure S12 ^{13}C NMR 1-methyl-5-(4-(methylsulfonyl)phenyl)-1H-imidazole (**3af**)

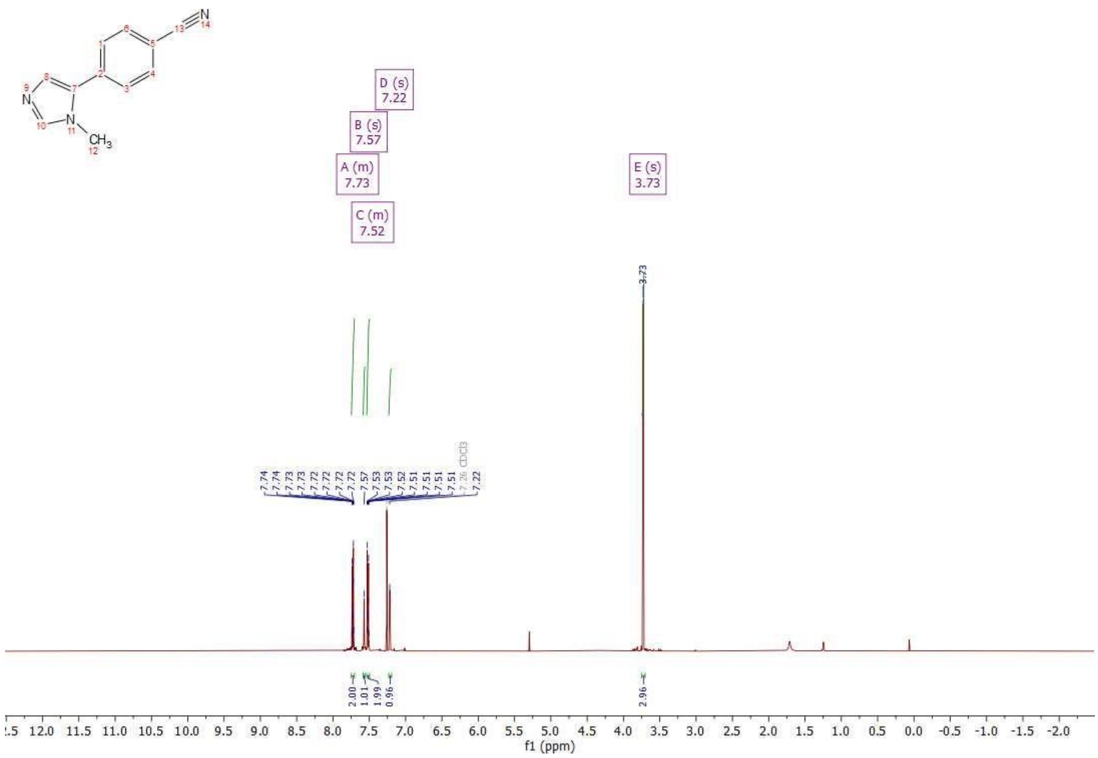

Figure S13 ^{1}H NMR 4-(1-methyl-1H-imidazol-5-yl)benzonitrile (**3ag**)

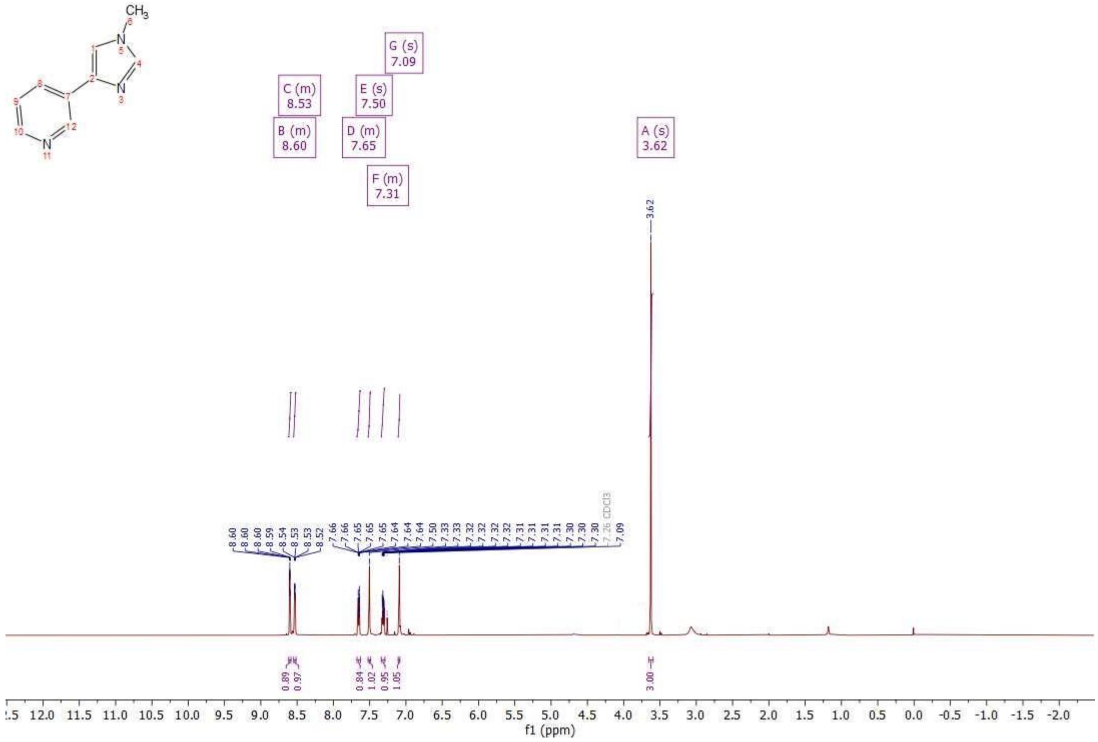

Figure S14 ^{1}H NMR 3-(1-methyl-1H-imidazol-5-yl)pyridine (**3ah**)

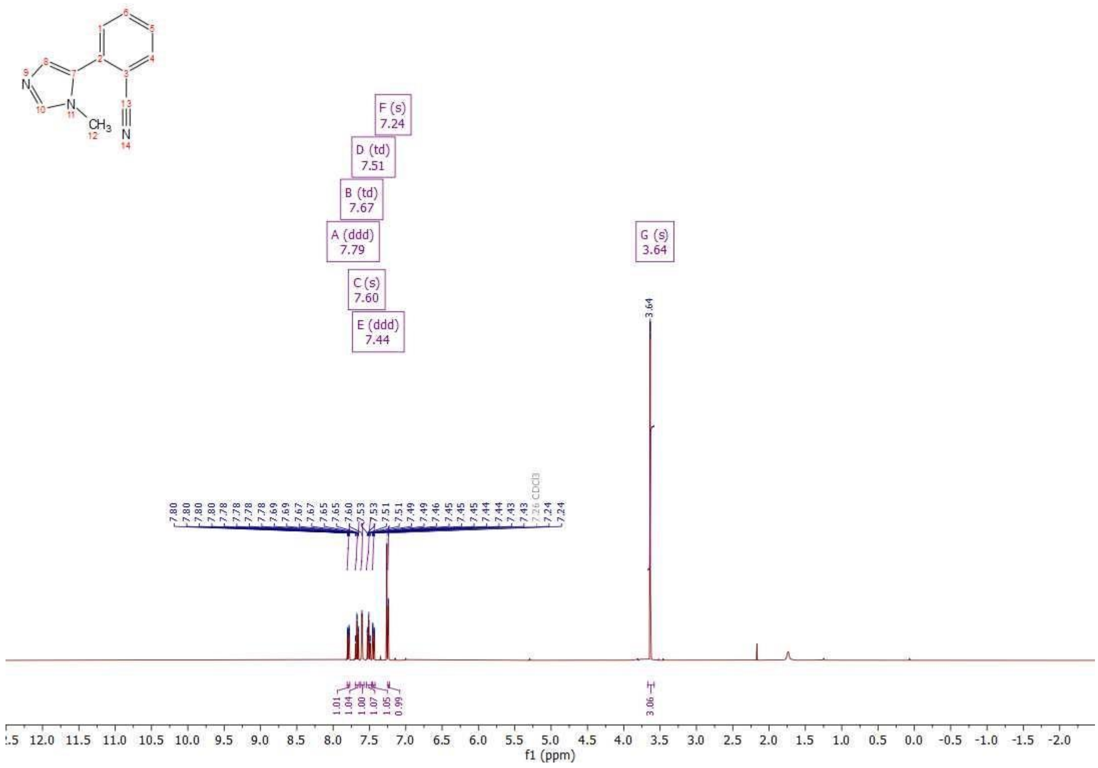

Figure S15 ^{1}H NMR 2-(1-methyl-1H-imidazol-5-yl)benzonitrile (**3ai**)

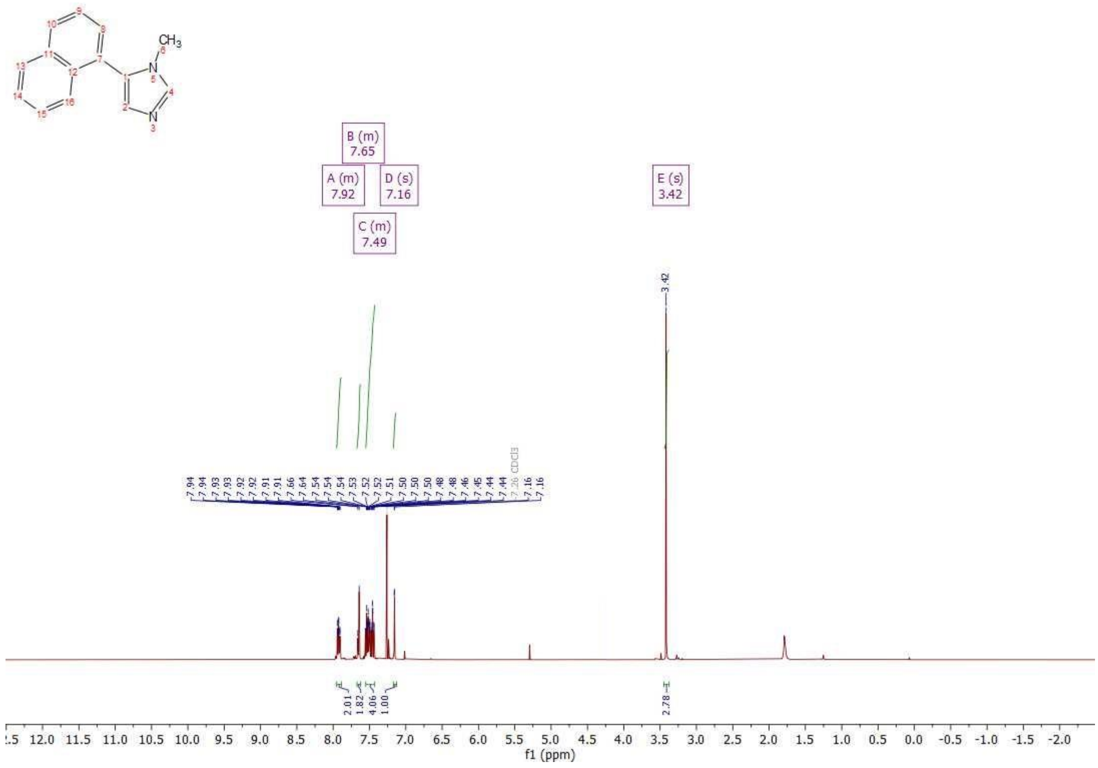

Figure S16 ^{1}H NMR 1-methyl-5-(naphthalen-1-yl)-1H-imidazole (**3aj**)
